# Emergence of biopharmaceutical innovators in China, India, Brazil, and South Africa as global competitors and collaborators

**DOI:** 10.1186/1478-4505-10-18

**Published:** 2012-06-06

**Authors:** Rahim Rezaie, Anita M McGahan, Sarah E Frew, Abdallah S Daar, Peter A Singer

**Affiliations:** 1 Munk School of Global Affairs, University of Toronto, Toronto, Canada; 2 Asia Pacific Foundation of Canada, Vancouver, BC, Canada; 3 Rotman School of Management, University of Toronto, Toronto, Canada; 4 TetraLogic Pharmaceuticals, Malvern, PA, USA; 5 Sandra Rotman Centre, University Health Network and University of Toronto, MaRS Centre, South Tower, 101 College Street, Suite 406, Toronto, ON, M5G 1L7, Canada

**Keywords:** Innovation, Emerging markets, Biotechnology, Pharmaceuticals, Biopharmaceuticals, Globalization, Entrepreneurship, Qualitative research

## Abstract

Biopharmaceutical innovation has had a profound health and economic impact globally. Developed countries have traditionally been the source of most innovations as well as the destination for the resulting economic and health benefits. As a result, most prior research on this sector has focused on developed countries. This paper seeks to fill the gap in research on emerging markets by analyzing factors that influence innovative activity in the indigenous biopharmaceutical sectors of China, India, Brazil, and South Africa. Using qualitative research methodologies, this paper a) shows how biopharmaceutical innovation is taking place within the entrepreneurial sectors of these emerging markets, b) identifies common challenges that indigenous entrepreneurs face, c) highlights the key role played by the state, and d) reveals that the transition to innovation by companies in the emerging markets is characterized by increased global integration. It suggests that biopharmaceutical innovators in emerging markets are capitalizing on opportunities to participate in the drug development value chain and thus developing capabilities and relationships for competing globally both with and against established companies headquartered in developed countries.

## Background

The UNESCO Science Report 2010 shows that the emerging markets of China, India and Brazil are, by many indications, leading most of the world in the pace of scientific and technological development. The growing research and development (R&D) expenditures and rising global share of scientific publications and patenting rates in these countries (see Table 1) reflect efforts by diverse actors to compete globally. National policies, domestic economic growth, and global trends over the last several decades have stimulated growth and innovation within domestic biopharmaceutical enterprises in China, India, Brazil and South Africa (which hereafter we call ‘emerging markets/economies’). Increasingly, this growth and innovation has led to unique advantages that allow some emerging-market firms to compete effectively on global markets rather than to serve primarily as suppliers, vendors and outsourcers to developed-country pharmaceutical multinational corporations. Growth in the pharmaceutical sectors of these four emerging countries continues to outpace that in developed countries (see Table 1). Brazilian and South African policy objectives have thus far focused primarily on import substitution and lowering the cost of health products for local populations. In contrast, those of India and China aim at nurturing an innovation ecosystem and a vibrant bio-economy with greater ambitions for exportation from the sector.

**Table 1:** Key R&D, publication, patenting, and pharmaceutical market indicators for China, India, Brazil, South Africa and select developed markets

**Country**	**R&D Expenditure as a % of GDP**^**1**^	**Gross Expenditure on R&D in Billions USD**^**1**^	**Share of Global Scientific Publications** **[Annual Growth Rate b/w 2000–2008]**^**2**^	**Annual Growth Rate in Patenting (2000–2006)**^**2**^	**Pharmaceutical Market Size, Billions USD, 2010 (Projected Size in 2015)**^**3**^	**CAGR for Pharmaceutical Market, 2006–2010 (2010–2015 Forecast)**^**3**^
**China**	1.5 (2008)	102	12%, [23.4%]	26.5%	25.7 (48.8)	17.3% (13.6%)
**India**	0.80	24.8	2.3%, [4.7%]**	42%^*^	14.1 (30.4)	16.6% (16.6%)
**Brazil**	1.10	20.3	1.6%, [12.2%]***	N/A	15.3 (34.4)	13.1% (17.6%)
**Canada**	1.84 (2008)	24.0	2.7%	3.9%	26.6 (30.2)	5.4% (2.6%)
**Germany**	2.54	72.2	4%08	5.7%	37.9 (41.5)	3.4% (1.8%)
**Japan**	3.44	148	4.8%	4.5%	72.4 (102.7)	4.4% (7.2%) ****
**United States**	2.82 (2008)	398	16%	2.6%	292.8 (344.7)	2.9% (3.3%)

Sources: ^1^ UNESCO Science Report 2010. Data is for 2007 unless stated otherwise.

^2^ OECD Science, Technology and Industry Outlook 2010. Share of publications is for 2008, except for India where this share represents 2006 Data.

^3^ DataMonitor 2010, Country-specific Pharmaceutical Industry Profile Reports.

* Estimate for India is for period 1997–2004.

** Estimate for India is for 1995–2005.

*** Estimate for Brazil is for 1998–2008.

**** Data for 2005–2009 and 2009–1014.

CAGR stands for Cumulative Annual Growth Rate.

While earlier research has focused on technology transfer through foreign direct investment, alliances, and other cross-border partnerships to boost developing-country entrepreneurship [1], indigenous innovation and entrepreneurship are receiving increasing attention as means of improving the health and wealth of the poor directly and for cultivating knowledge capabilities in resource-limited settings [2]. Existing research shows that the global pharmaceutical multinational corporations (MNCs) based in resource-rich countries have tended not to develop drugs for exclusive use in the developing world or to make significant investments in drug development for neglected diseases [3-5]. The role of emerging market firms in filling this gap, as well as enhancing affordability, is of much interest, but is not a key focus for this paper. Suffice it to say that previous publications [6], and our ongoing studies, do suggest that emerging market firms, as a group, have a greater overall focus on addressing developing-world diseases than their major global competitors. Nonetheless, we have also argued [7] that diseases that almost exclusively affect poor market segments in the developing world are unlikely to be addressed by emerging market firms as well, and require specific attention from the global health community and local and international governments.

This paper analyzes key issues and implications of corporate innovation in emerging economies. The core research questions we address here are: how do health technologies develop in emerging economies? Have companies in emerging economies specialized on technologies? What entrepreneurial strategies and enterprise structures have been commonly used to facilitate the transition into business models that emphasize innovation? What are the key barriers to health product innovation? What is the role of the state in innovation within health enterprises in the emerging markets? What is the role of emerging market companies in the global pharmaceutical sector, and how do emerging-market competitors interact with incumbent firms based in the developed economies?

The results of our analysis of the mechanisms that shaped corporate competitiveness in emerging markets yields insights at two levels: first, on country policies that enable and support technological upgrading and specialization; and second, on entrepreneurial strategies and business models that enhance corporate innovation and global competitiveness.

## Methods

The basis of this article is qualitative case study analysis of indigenous biopharmaceutical enterprises in each of Brazil, China, India and South Africa. Qualitative research approach was chosen as it can help us understand a phenomenon of interest in its complexity, to identify areas that need to be influenced, and to see the consequences of policy intervention in real life ([8]; p.10). This analysis builds on a series of country-specific studies published in the *Nature Biotechnology* journal [9-12], which focused on products and services, partnerships, intellectual property (IP) portfolios, business models, financial environments and barriers to development for companies in each country. This analysis is a comparative assessment across the four nations and is informed by face-to-face interviews with representatives of 91 domestic biopharmaceutical companies (Table 2) as well as 25 other institutions in the stated countries. Institutional informants included government agencies involved in biopharmaceutical development, venture capital firms, technology parks/incubators, and industry associations. The interview data complemented insights gained from published reports and articles, websites of companies and relevant government institutions and policy documents. Interview data was employed to enhance confirmation of and elaboration upon documentary evidence. The firms chosen were primarily involved in the development and production of vaccines, therapeutics, and diagnostics. With a few exceptions, interview data was collected through site visits and semi-structured, face-to-face interviews lasting approximately 60–90 minutes.

**Table 2:** Health biotech firms from China, India, Brazil and South Africa included for this analysis

**China**	**India**	**Brazil**	**South Africa**
Amoytop Biotech	Avestha Gengraine Technologies	Aché Laboratórios Farmacêuticos	African Clinical Research Organization (ACRO)
Beijing Haiyan Pharmaceutical	Advinus Therapeutics	BioCancer	Altis Biologics
Beijing Wantai Biological Pharmacy Enterprise	Bharat Biotech International	Biolab Sanus Farmacêutica	AngioDesign
Bio-Bridge Science	Bhat Bio-Tech India	BioGene	Arvir Technologies
CapitalBio Corporation	Bharat Serums andVaccines	BIOMM Technology	Aspen Pharmacare
FusoGen Pharmaceuticals	BigTec Laboratories	COINFAR	Bioclones
GeneScience Pharmaceuticals	Biocon	Cryopraxis Criobiologia	Biomox
Hutchison MediPharma	Biological E	Eurofarma Laboratórios	Biovac Institute
HD Biosciences	Dr. Reddy’s Laboratories	FK Biotecnologia	Cape Kingdom
Kanghong Pharmaceutical Group	GangaGen Biotechnologies	Hebron Farmacêutica	Disa Vascular
Shanghai Ambrosia Pharmaceutical	Indian Immunologicals	Intrials Clinical Research	Elevation Biotech
Shanghai Fudan-Yueda Bio-Tech	Jubilant BioSys	Katal Biotecnológica	Gknowmix
Shanghai Genomics	LifeCare Innovations	Labtest Diagnóstica	Kapa Biotech
Shanghai Genon Bio-Engineering	Lupin Pharma	Nortec Química	National Bioproducts Institute
Shanghai Huaguan Biochip	Piramal Life Sciences	Pele Nova Biotecnologia	Synexa Life Sciences
Shanghai Sunway Biotech.	Panacea Biotec	Recepta Biopharma	Veritrial Clinical Trials
Shanghai United Cell Biotech	Reliance Life Sciences	Scylla Bioinformatics	Vision Biotech
Shenzhen Beike Biotechnologies	Serum Institute of India	Silvestre Laboratories	
Shenzhen Chipscreen Biosciences.	Shantha Biotechnics	União Química	
Shenzhen Tiandakang Gene Engineering	Strand Life Sciences		
SiBiono GeneTech	Suven Life Sciences		
Simcere Pharmaceutical Group	Transgene Biotek		
SinoCells Biotech	Wockhardt		
SinoGenoMax	Clinigene		
Sinovac Biotech	SIRO Clinpharm		
Starvax International	Syngene		
Tianjin SinoBiotech			
WuXi PharmaTech			
Zensun (Shanghai) Sci. &Tech. Co., Ltd.			

## Results and discussion

### Health technology innovation and the emerging markets

The modern pharmaceutical industry finds its origins in the synthetic dye industry in Germany and Switzerland and the discovery of medicinal effects of some dyestuffs [13]. The large-scale production of penicillin during World War II spawned considerable R&D investments and productivity in terms of pharmaceutical innovations particularly by the major US-based companies [13]. The 1976 inception of Genentech (San Francisco, now part of Roche based in Basel, Switzerland) spawned the modern biotechnology industry. The latter is characterized by many small- and medium-sized enterprises (SMEs). With the exception of a few large firms, biotech SMEs are dispersed across a considerable number of countries. Notwithstanding somewhat distinct developmental trajectories, pharmaceutical and health biotechnology sectors are increasingly intertwined due to mergers and acquisitions and overlapping activities. While key firms remain based in a relatively small group of countries – including the U.S., the United Kingdom, Switzerland, Germany, and France – the global dispersion of their activities is increasing, as is the emergence of indigenous firms in different countries.

The geographic concentration of innovative activities in health technology is thought to be a function of historical differences in institutional environments [13]. Therefore, it is of interest to examine how the changing institutional context in the emerging markets has impacted the development of biopharmaceutical sectors in these nations. The adoption of the World Trade Organization’s Trade-related Aspects of Intellectual Property Rights (TRIPS) agreement has been among key institutional adjustments and has enhanced the impetus to innovate. It is against this backdrop that we witness a growing number of firms in the emerging markets building innovative capability. This capacity may be particularly crucial in the post-TRIPS era where the power of emerging market manufacturers to affect drug prices in poor markets is likely to be diminished [14], and drug prices rise due to the marketing of high priced products by pharmaceutical MNCs [15].

#### Innovation, technological upgrading and industry specialization

The health biotech sectors of emerging economies are undergoing a major transformation characterized by increased technological sophistication of product portfolios and R&D activities. In a recent study, we identified 376 medicinal or vaccine candidates within the pipelines of 66 indigenous companies in China, India and Brazil (Unpublished Results). An estimated 60% of the 376 candidates involve new chemical or molecular entities, and as such could be considered new-to-the-world type of innovations. The rest include innovations that build on known molecules or products. Among the 376 candidates identified, over two-thirds are in the discovery or preclinical stage, and only about 3% (11) of products have reached the domestic market. These results, together with studies cited previously, demonstrate that domestic companies in the emerging markets are developing new capabilities and novel health products, but also that they are at a relatively early stage from the perspective of innovation.

A common strategy for entrepreneurs in emerging economies has been to develop technologically and financially less demanding products before venturing into more sophisticated areas as internal capabilities and revenues improve. Historically the major thrust of firms in emerging economies has been mainly to offer non-novel products and services under contract to established pharmaceutical multinational firms, or as low-technology traditional formulations. By and large, the development of globally innovative products by emerging market firms is a relatively recent phenomenon and has been enabled by a number of factors. Reverse engineering of existing drugs has had a considerable learning effect for industries in China, India and Brazil, facilitating the adoption of new technologies and easing the transition into innovative activities. Firms have not only gained experience in health product manufacturing and marketing, but have also developed the necessary technical expertise to allow them to venture into more sophisticated areas. In this respect, the absence of a strong domestic pharmaceutical manufacturing sector in South Africa puts it in a different category than the other three countries studied, resulting in the dearth of a skilled knowledge base on which to build.

A key driver of technological upgrading, particularly by Indian firms, has been an interest in export markets, including the major markets of the U.S. and Europe. While domestic populations constitute the main market – at least presently – for most health biotech enterprises studied, there is a growing interest among executives in many medium and large firms to export products. Thus far, export of finished products is notable only in India, where the traditional pharmaceutical sector has increased its foreign sales by over 21% annually between 1996 and 2005 [16] and the country’s fast-growing biotech sector, as a whole, garners 56% of its revenues from exports [17]. Major Indian firms have been able to increase exports to the U.S. and elsewhere, by upgrading their manufacturing facilities to meet the U.S. Food and Drug Administration (FDA) requirements. The growing number of strategic alliances between pharmaceutical MNCs and Indian companies also allows the latter group of firms to capitalize on their manufacturing capacity and the considerable marketing resources of MNCs to address global markets [15]. In contrast, few companies in China, Brazil, and South Africa have thus far exported finished medicines to highly regulated markets. This trend is likely to change however, as these countries continue to enhance their own manufacturing standards.

Notwithstanding the fact that health biotech industries in the emerging markets are in a transition period, one can observe patters of specialization by country. Diagnostics and medical devices were common starting points for a number of firms in Brazil and South Africa, respectively, while vaccines represent a major entry point for many firms in India. For instance, Hyderabad-based firms Bharat Biotech and Shantha Biotechnics (now part of Paris-based Sanofi-Aventis) started out in vaccine manufacturing, and have since expanded their range of activities to include manufacturing of other recombinant products. China’s Beijing Wantai, primarily a diagnostics company, has focused on innovative research including efforts to commercialize a novel hepatitis E vaccine. Similarly, Brazil’s FK Biotech (Porto Alegre) has expanded its range of activities from manufacturing of monoclonal antibodies for diagnostic kits to development of cancer vaccines. Starting with local markets, some firms have become major players at a global scale. Vision Biotech (Cape Town, South Africa) for example claimed to be the second-largest manufacturer of rapid tests for malaria worldwide, and the Serum Institute of India (Pune) is a major global vaccine supplier. Also, a growing number of incumbent pharmaceutical companies are developing manufacturing capabilities for recombinant products, with many in India and China already benefiting from related efforts over the past decade.

Indian companies have become key global vaccine suppliers. A dozen vaccine companies generate over 50% of the annual revenues for the biotech sector, a market that reached US$2.5 billion in 2007/8 [17]. The entry of Indian, as well as some other developing-country firms, into vaccine production took off in the 1990s as manufacturers in the industrialized countries began to abandon these markets in favor of more lucrative ones elsewhere [18]. It has been suggested that the emergence of Global Alliance for Vaccines and Immunization (GAVI) and the GAVI Fund played a significant role in stimulating the technological expansion of developing world vaccine manufacturers particularly in India and Brazil [18]. Among the largest Indian vaccine producers are Serum Institute of India, Panacea Biotec (New Delhi), Biological E (Hyderabad), Bharat Biotech and Shantha Biotechnics. Some of these firms have become major global suppliers of vaccines to public immunization programs in collaboration with the UNICEF, PAHO and the WHO [11]. Recent involvement of Indian companies - namely, Bharat Biotech, Zydus Cadila (Hyderabad), Panacea Biotech, the Serum Institute and Biological E - in the race towards a domestically developed vaccine for H1N1 influenza signals the country’s growing technological capability in vaccine development. It is worth mentioning that some of these firms relied on in-licensing of the core technology from foreign firms. Other key focus areas for the Indian firms include a growing trend towards production of biogenerics and the discovery and development of novel small-molecule drug candidates. Mumbai-based Piramal Life Sciences and Glenmark Pharmaceuticals are among the leaders in this group, each having over 10 lead drug candidates in development. Other Indian firms are working on developing new drug delivery systems, monoclonal antibodies and diagnostic tests, and manufacturing recombinant medicines [11]. In anticipation of India’s adoption of the TRIPS agreement in 2005, the pharmaceutical industry in India spent approximately US$292 million (13.2 billion Indian rupees) on R&D between 2003–2004, accounting for 3.6% of overall revenues [19]. The global competitiveness of India’s private industry in both vaccines and therapeutics is, in part, a result of the relative openness of its domestic market and limited involvement of the government sector in health product manufacturing. In China and Brazil (and until recently in South Africa), the prominent role of state-sponsored or public sector institutes in vaccine development and production has diminished the scope for the domestic private sector in this area.

Chinese firms involved in innovation have a considerable focus on developing novel therapeutics in frontier areas and often leverage traditional Chinese medicine (TCM) knowledge and resources. Gene therapies against some cancers, developed by Shenzhen SiBiono (Shenzhen) and Shanghai Sunway (Shanghai), have been on the market for over four years [10]. As of mid-2009, approximately 9,000 patients had been treated for head and neck cancer with SiBiono’s innovative gene therapy product, marketed as Gendicine®, approximately 1,200 of whom came from outside China. Other innovative projects in the pipeline included a novel drug candidate for cutaneous T-Cell lymphoma by Shenzhen Chipscreen (Shenzhen), an HIV treatment candidate by Fusogen (Tianjin) and products to address lung fibrosis and liver cirrhosis by Shanghai Genomics. There are also significant efforts towards modernization of TCM, involving manufacturing quality control, efficacy tests, and determining active ingredients and their mechanisms of action. While China’s efforts towards new vaccine development have generally trailed behind India, strong emphasis by the Chinese government in recent years on technological advancement in this area may be reversing this trend. The Chinese vaccine-manufacturing sector is one of the largest in the world in terms of production volume, where approximately 30 vaccine manufacturers, mostly domestic enterprises, produce over a billion vaccine doses annually [20]. As a sign of recent progress, Sinovac Biotech (Beijing) gained manufacturing approval in September 2009 from the Chinese State Food and Drug Administration (SFDA) for its single-dose H1N1 vaccine – a time frame similar to leading global firms and preceding the launch of clinical trials by its Indian counterparts mentioned above.

The Brazilian health biotech industry is populated by a growing number of technology-based small and medium sized (SME) enterprises as well as generics-based pharmaceutical incumbents, some of whom are transitioning to innovative R&D activities. Overall, the sector remains very young with the vast majority of health biotech enterprises coming into existence in the past decade [21]. A handful of the SMEs we studied had marketed and/or were developing novel technologies. Examples of these firms include Pele Nova Biotechnologia, Recepta Biopharma (both in São Paulo), and FK Biotec (Porto Alegre). Some of these firms have leveraged early success in diagnostics to develop more technologically advanced products [9]. Similarly, large generics firms including Cristália (Itapira) and Aché Laboratories (São Paulo) are building capabilities in discovery and development of innovative drugs. Some firms such as Aché and Eurofarma (São Paulo) were developing capabilities to manufacture large molecules, as part of their foray into biotechnology. However, regardless of size, Brazilian companies tend to shy away from vaccine development and production due to the dominant role played by the country’s public sector in this area. Furthermore, in Brazil and China the public sector supplies a considerable portion of basic medicines deemed essential for public health. Where public and private domains have significant, overlapping, and unclear boundaries it can create tension between the sectors [9], possibly detracting from the country’s overall innovative capacity.

The nascent health biotech sector in South Africa relies considerably on the research capabilities of the country’s universities and research institutes to identify novel technologies and products for commercial development [12]. For example, Elevation Biotech (Johannesburg), which was spun out of the University of Witwatersrand and the National Health Laboratory Service (both in Johannesburg), is developing novel peptides to inhibit HIV entry into cells. Elevation has received grants from the South African government and the International AIDS Vaccine Initiative (New York) to develop and test new vaccine antigens. Other innovative firms include: iThemba Pharmaceuticals (Modderfontein) focused on drug development for tuberculosis (TB), malaria and HIV/AIDS, Arvir Technologies (Modderfontein) is developing cost-effective methods to manufacture HIV drugs, and Disa Vascular (Cape Town), which is involved in development and manufacturing of coronary artery stents. A few South African firms have also ventured into nutraceuticals including Biomox Pharmaceuticals (Pretoria) and Cape Kingdom (Cape Town). Biovac Institute (Cape Town) was founded in 2003 as a public-private partnership to supply South Africa’s vaccination program, after attempts at a purely public system failed to accomplish this goal. It remains the only vaccine manufacturer in Sub-Saharan Africa.

In all four countries, locally abundant natural resources are increasingly leveraged to identify and develop novel health technologies. Biodiversity resources and traditional medicines serve as a major source for lead compound identification for firms in the emerging markets. For example, Shanghai Ambrosia Pharmaceuticals (Shanghai) uses its novel platform technologies to screen TCMs for lead molecules against cancer and immunological diseases. Brazil’s Aché Pharmaceuticals and Pele Nova Biotechnologia have marketed phytotherapeutic-based products used for inflammation and skin lesions respectively, and a number of others are developing other products originating from Brazilian biodiversity [9]. India’s Piramal Life Sciences, in collaboration with public universities and research institutes, has developed a large bank of natural products and product extracts as well as a microbial library containing over 40,000 different cultures. Many of the company’s lead molecules under study – including some in clinical trials – were sourced from these assets. Avesthagen (Bangalore) uses a multi-disciplinary approach to develop health products, including dietary supplements, drawing extensively on knowledge from Indian medicinal plants.

Specialization patterns are broadly reflective of a complex interplay involving the institutional environment in each country, local knowledge as embodied in universities and research institutes, access to unique and country-specific resources, the divergent involvement of the public sector in health product manufacturing and provision, and the evolving competition and cooperation between local and global rivals.

#### Entrepreneurial innovation strategies and business models

As recently as a decade ago, life-sciences firms in emerging markets were engaged mainly in activities such as manufacturing, formulating, packaging and distributing generic products. By contrast, today a much broader scope of business models and strategies are used to generate revenues and invest for future prosperity across the emerging markets. A growing number of firms view product R&D as an important component of their business models. However, the extent of their commitment to R&D and the nature of their involvement vary across firms and countries. The divergent involvement in innovation is influenced by two key factors. Global MNCs have historically tended to concentrate in high margin market segments in the emerging markets, leaving domestic companies to settle for second and third tier cities, rural areas and other neglected niche segments. The resulting pressure on margins for local companies, together with dearth of private capital to support biopharmaceutical innovation has meant that indigenous firms have also had to innovate in business models.

First, the relatively few pure R&D firms in existence today, such as Recepta BioPharma (Brazil), Fusogen Pharmaceuticals Inc. (China), and iThemba Pharmaceuticals (South Africa), have tended to garner significant support from governmental sources. Most companies we studied in China, Brazil and South Africa had received some government funding for their innovation projects. Indian firms have traditionally had less direct support from government sources, perhaps contributing to the near absence of a pure R&D-based business model among firms in this country.

Second, health enterprises studied rely considerably on partnerships with domestic universities and research institutes as well as foreign entities. Firm-university linkages typically serve to fill in gaps in internal R&D capabilities and access facilities/equipment and, to a lesser extent, to the transfer of new technologies to firms. Notwithstanding these benefits domestic university-company links remain weak overall, largely for cultural and historical reasons. There appears to be a strong correlation between the degree of firms’ engagement in innovative R&D and their likelihood of having linkages with domestic universities and research institutes in all countries studied. For example, R&D-intensive firms such as Recepta Biopharma of Brazil, Shanghai Sunway and Sinovac Biotech of China, and Avesthagen in India have strong collaborations with academic institutions. Similarly, most South African firms, which are often established to commercialize technologies originating from universities, maintain close linkages with these institutes. In contrast, domestic entrepreneurial collaboration, particularly partnerships aimed at product development, appears limited across all countries studied.

Third, most innovation-minded entrepreneurs have adopted an indigenous growth model at or shortly after inception, where R&D activities are financed through internal revenue generation. The result is that these enterprises often undertake two distinct sets of activities: one set aims at generating revenues in the short-term, while the other is focused on innovation for long-term growth. For example, when Mr. Eduardo Cruz, the founding director of Cryopraxis (Rio de Janeiro), wished to start a company in 2000 that would develop stem-cell therapies, his initial strategy was to start a cord blood banking service to help finance R&D activities. A common approach for generating revenues in all four countries remains the in-house manufacturing and marketing of products, be they generics, copycat therapeutics, or modifications of existing health products often targeted at niche markets. Another prevalent strategy among innovative firms in China and India is to offer a variety of product development and/or manufacturing services, often to foreign clients. Examples of firms that grew by utilizing this model include India’s Bharat Biotech and the Chinese firms Shenzhen Chipscreen and Shanghai Genomics. In industrialized economies it is well established that financing new product development with current revenues is more expensive and constraining compared to investment-driven financing. Nonetheless, even where available, emerging market equity investors typically demand revenues from early on in Investee Company’s life cycle – in some cases causing them to abandon innovative projects with extended development periods.

Fourth, a relatively recent trend particularly notable in China and India is growing international linkages of domestic firms to advance innovative R&D activities. These partnerships often manifest in one of two forms – outsourcing or co-development arrangements. The provision of contract R&D services in the emerging markets is relatively new and transactional in that services are rendered in return for payments upon completion of specified milestones. Previously, domestic companies only offered manufacturing and clinical research services with limited capability in other aspects of drug discovery and development. Companies adopting the newer model typically serve foreign clients by focusing on discovery and development as well as contract manufacturing for proprietary health products. Among a growing group of service providers are China’s Wuxi Pharmatech, Sundia Meditech Company (both in Shanghai) and India’s Advinus Therapeutics (Bangalore) and Jubilant Organosys (Noida), all of whom have enjoyed considerable growth in recent years. Co-development projects on the other hand involve joint discovery and/or development of novel technologies between Indian and Chinese firms and foreign counterparts, usually large multinational corporations (MNCs). China’s Hutchison MediPharma (Shanghai) is an integrated R&D company whose discovery and development activities are supported, in part, through relationships with Eli Lilly (Indianapolis, IN), Merck KGaA (Whitehouse Station, NJ) and Johnson & Johnson’s subsidiary Ortho-McNeil-Janssen Pharmaceuticals (Raritan, NJ). India’s Jubilant Biosys (Bangalore) and Suven Life Sciences (Hyderabad) are other examples of companies that have significant levels of collaborations with multinational firms. What distinguishes co-development from outsourcing is that under the former approach, the emerging-markets partner shares in longer-term risks and returns form commercialization projects. Such relationships are motivated, in part, by the desire on the part of multinationals to reduce drug development costs by tapping cheaper scientific labor in the emerging markets. Benefits to domestic firms include: access to financing for innovative projects, technological learning, sharing of future royalties, and reputational advantages of working closely with major global enterprises.

### Barriers to innovation

Our analysis revealed a number of important barriers that hinder entrepreneurial progress in health product innovation in the countries studied. Although many of the challenges are similar across the four countries studied, the underlying mechanisms that give rise to the challenges differ.

#### Human resources

While all four countries have expanding capabilities in science and technology in general and life sciences in particular, access to specially trained personnel in health biotech innovation is often limited. The most common strategies to fill shortage of skilled labor have been to: a) increase science and technology capacity in general through investment in R&D, b) develop targeted training programs, and c) recruit highly trained personnel from abroad. As a result of the first strategy, all countries studied have increased their relative contribution to global scientific publications in recent years. In particular, China’s publication and citation rates have been rising substantially, making it the fifth largest publisher globally [22]. This country’s scientific publications grew at an average annual rate of 16.5% between 1995–2005, compared with 4.7 and 8% for India and Brazil respectively [23]. While this trend speaks to a strengthening of the science and technology base, it is often not sufficient to address shortages of specially trained personnel. Efforts by the emerging economies to train personnel domestically are not only time-consuming, but face challenges of their own. Many training programs relevant to health biotech in the emerging economies are based in university systems, which have been traditionally isolated from industry. As a result they are not perceived to be effective at detecting and addressing the changing industrial demands.

Nonetheless, some efforts in this regard are underway. For example, India’s Department of Biotechnology has a number of programs including sponsorship of post-graduate training in biotechnology and the provision of stipends for post-graduate students to work in the industry for six months. Similarly, Brazil has initiated a program that sponsors researchers’ salaries for a period of time while they work in the industry. South Africa’s human resource challenges seem related to the country’s inability to sufficiently retain highly trained individuals [12] and the lack of a strong pharmaceutical manufacturing sector to provide a basic skill-set for further industrial development. Lastly, the Chinese, and to a lesser extent Indian, industries have been beneficiaries of returnees, who in many cases have obtained specialized training abroad and have strong scientific and commercial networks. China’s post-1978 policies towards overseas study allowed tens of thousands of Chinese students and scholars to go abroad for education and training. However, only an estimated 25% of these students return to China after their training [24]. In response, the Chinese government has put in place an ambitious suite of programs and incentives to recruit back highly-trained Chinese professionals and academics from abroad, with mixed results [24]. For instance, Simon and Cao find that these incentives have thus far failed to recruit the most-highly trained and accomplished groups [24]. What is also uncertain is whether those who do return will be sufficiently supported to stay for the long term, and if the skewed compensation scheme in favor of returnees can be maintained without adversely affecting overall employee morale. What is clear is that China’s ambitious R&D infrastructure-building programs, as part of its overall goal to build an innovative and globally competitive bioeconomy, is likely to create ongoing demands for specialized skills in various areas. It remains to be seen whether current initiatives will be sufficient to meet this demand.

#### Access to R&D infrastructure

As many companies in the emerging economies have ventured into innovative activities only in recent years, their in-house R&D capabilities and infrastructure are often limited. A common strategy to obtain required services is to partner with researchers within universities and research institutes. This strategy is helpful to a degree but a number of factors detract from its effectiveness. These include the historical lack of an entrepreneurial culture within universities and the traditional isolation of the public and private sectors in these countries. Although governments have made some efforts to bridge the divide between public and private sectors – often through funding of collaborative projects – in most cases there is little indication that these efforts have made a significant impact. An exception to this may be China, where the involvement of government in many state-owned enterprises (SOEs) is thought to make interactions with universities and research institutes easier for these firms. In addition, China has embarked on an ambitious infrastructure-building exercise through its Hi-tech Parks Initiative. In the span of less than 20 years, the country has built 54 high-tech parks, an estimated 20 of which have a life sciences component. The largest of these is the Zhangjiang Hi-Tech Park in Shanghai. Initiated in 1992, this park occupies an area of 25 square kilometers and is already home to over 4,000 enterprises, with approximately 400 being life science companies, institutes and service providers. Other major life science clusters include Zhongguancun Life Science Park in Beijing, and bioBay in Suzhou, which is a joint project between China and Singapore. These parks offer a host of benefits and invariably have one or more incubator facilities, which especially cater to small and medium technology-based enterprises. While India and Brazil have a few clusters, that are similar in certain respects, the scale and speed of development in China is breathtaking by comparison.

#### Regulatory issues

One of the most common challenges facing innovation-inspired firms in the emerging economies relates to the clarity and effective enforcement of regulations governing health products. Again, while the outcome often manifests in delays in regulatory approval, the underlying causes often vary across nations. For instance, while a fragmented regulatory regime in India has historically posed serious challenges to the growth of the innovative health biotech sector, lack of practical experience on the part of Brazilian and South African regulators was thought to make product approval challenging in these countries. Other challenges in Brazil and South Africa were primarily related to delays in approval of clinical trials, which was perceived to detract from a major competitive advantage possessed by these countries. In recent years India has been in the process of reforming its biotech regulatory system by trying to institute a centralized regulatory agency called the National Biotechnology Regulatory Authority (NBRA). Long in coming and motivated in part by the desire to better regulate genetically modified crops, limitations on NBRA’s scope of activities serve to maintain the anachronistic separation of institutions that govern small- versus large-molecule drugs. In this sense, India is missing the opportunity to create a truly centralized regulatory agency, which can oversee all heath products and optimize resultant synergies. China’s regulatory regime has also had significant setbacks in recent years due to corruption scandals involving some high-ranking officials at the country’s SFDA. However, the agency has rebounded from these challenges and has recently made progress in a number of areas. For example, in early 2009 the SFDA instituted its Green Channel initiative, which is an expedited approval process, allowing regulators to waive requirements for Chinese trials for innovative new drugs, new combinations, and those targeted at unmet medical needs in the country. Furthermore, it has undertaken a variety of initiatives aimed at addressing product quality issues in the country’s vast and complex pharmaceutical sector.

#### Intellectual property protection

The theoretical rationale underlying patenting is that it incentivizes innovation efforts by creating a temporary monopoly. The TRIPS agreement was an attempt to expand the number of countries and product classes where such markets could be established. Although evidence on the impact of strong IP protection on innovation efforts of firms in all sectors is mixed [25-31], the relationship has been much more firmly established in the pharmaceutical sector [32], albeit not in all countries [33]. This is, in part, due to the fact that once discovered pharmaceutical products/molecules are relatively easy to copy and reproduce [34]. While it is generally believed that IP protection incentivizes greater investments into innovative R&D activities, it is not the only driver of R&D expenditures. Market demand [35,36], and enhanced market valuation [37] are among other factors that tend to motivate firm-based R&D.

While biopharmaceutical firms in all four countries are increasingly cognizant of the importance of patenting, issues related to the efficiency of processing patent applications and effective enforcement of property rights continue to create uncertainty. In all of the countries studied except South Africa, a new pharmaceutical product patent regime was formally adopted between 1993 and 2005. While these countries are still adjusting to the new IP regime, they also struggle to minimize the negative impacts it might have for both public health and domestic industries. These attempts have resulted in country-specific peculiarities in patent legislation that serve to diminish associated incentives and have led to a few high-profile court cases – particularly in Brazil and India where patent rights have either been denied or not enforced as expected. Among the most controversial elements built into patent legislation are provisions related to compulsory-licensing and those that aim to curtail the practice of ‘ever-greening’, which allows companies to collect monopoly rents by patenting minor changes to a given drug whose patent life is about to expire. For instance, in September 2009, the Indian patent office rejected an application by Gilead Biosciences (Foster City, USA) for the anti-AIDS drug tenofovir, in part based on arguments that the application lacked sufficient inventiveness. Brazil had earlier declined patent rights for the same product, declaring it of interest to public health in that country. Under the new Indian patent act, new uses for existing products are not patentable unless they demonstrate improved efficacy [18]. It is not yet clear how this will impact domestic companies, many of which are mainly concerned with improving existing technologies such as combination vaccines and new drug-delivery systems. Concerns regarding China’s patent regime stem primarily from the nature of its decentralized enforcement regime. Patent infringement cases are generally handled by municipal courts at the jurisdiction of the accused, introducing potential conflicts of interest into the process [38]. Amendments to China’s patent legislation, which took effect in October 2009, are aimed at enhancing the quality of patents, improving enforcement and alignment of China’s approach with that of other major markets.

#### Innovation financing

While private financing, especially from institutional investors, has been improving in the emerging markets in recent years, relatively few of these investors back companies that undertake highly innovative projects. Venture capital (VC) firms that have made investment in innovative companies include BioVeda China Fund (Shanghai), APIDC Ventureast Biotechnology Venture Fund (Hyderabad, India), DFJ-FIR Capital (Belo Horizonte, Brazil) and BioVentures (Cape Town, South Africa). Limitations on financial exits for investors are thought to be a key contributor to the shortage of VC investments in innovative companies. The BOVESPA-MAIS (São Paulo) branch of the Brazilian stock exchange and China’s recently launched Shenzhen Stock Exchange aim at improving the investment environment for technology-based companies. On rare occasions, they have listed in foreign stock exchanges as a way to achieve a more favorable valuation. Recent interest in company acquisition by multinational corporations in China and India may provide yet another avenue for investors to divest, and may eventually attract more investors into the sector. Volatility of stock markets, highly exaggerated price to earning ratios, and lack of sophisticated secondary markets are all detrimental to financing of high-risk innovation projects. The role of debt financing and the need to attract foreign capital in non-debt forms are crucial in the context of emerging markets. In the meantime, many companies in the emerging markets, particularly the small and medium enterprises, rely considerably on governmental support to finance innovation projects.

### Role of the state in advancing innovation

Overall commitment to science and technology development, as measured by national R&D expenditure, has been growing in all four emerging markets studied. China’s R&D expenditures as a portion of its gross domestic product (GDP) was 1.44% in 2007, amounting to approximately US$102 billion in purchasing power parity (PPP) – a more than doubling from its US$39 billion expenditure in 2002 [39]. While India and Brazil’s expenditures remained fairly constant as a portion of GDP (at 0.78% for India and 1% for Brazil), their real R&D investments nearly doubled during the 2002–2007 period due to GDP growth – rising from approximately USD 13 to 25 billion in India and US$13 to 20 billion in Brazil in PPP [40,41]. South Africa doubled its R&D expenditures between 1997 and 2005 when its overall R&D expenditures were 0.9% of GDP, approximately 53% of which was contributed by the business sector [42]. In contrast, businesses R&D expenditures as a percentage of national expenditures constituted 29.6% in India, 44.7% in Brazil, and 70.4% in China in 2007 [43].

Among the countries studied, China has the largest pool of public money dedicated to its science and technology programs. It administers much of these funds through three key programs: the High-Tech Research Development Program (known as the 863 Program), the Torch Program, and the National Key Basic Research Program (known as the 973 Program). The Torch Program is perhaps of most direct significance to the industry as the other two primarily focus on basic research. This program has an array of initiatives designed to support development of new technology industries. Since its inception in 1988, it has facilitated the building of 54 National High-tech Parks, which are designed to integrate R&D, manufacturing and enterprise incubation. The Torch program also administers a 7 billion RMB (US$1.02 Billion) innovation fund (Innofund), which funded 11,980 firms from 1999–2007, with approximately 20% of funds devoted to biotechnology. China’s 12^th^ Five-year Plan (2011–15) had dedicated US$308 billion to science and technology development with biotechnology as a priority sector for advancement.

India’s Department of Biotechnology (DBT) is the lead government agency that promotes biotechnology and pharmaceutical innovation in the country. DBT targets approximately one third of its annual budget – estimated at approximately Rs. 900 Crores (~US$201 M) for 2008/9 – to promoting public-private collaborations. Among its key programs supporting industrial R&D is the Small Business Innovation Research Initiative (SBIRI), which in 2009 provided funding for 48 projects within enterprises. The DBT’s other key foci are in skill development and regulatory reform. The overall contribution from other agencies, particularly with respect to support of R&D within enterprises has been more modest thus far.

In Brazil, the Studies and Projects Funding (FINEP) Program of the Ministry of Science and Technology is the key program that supports enterprise R&D. Through a series of initiatives, including sector-specific funds and investments in venture capital funds, FINEP aims to advance innovation within the industrial sector. One of its latest initiatives is the Prime Program (Primeira Empresa Inovadora), which aims at supporting approximately 5,000 technology-based startup companies between 2009–2011. It offers R$120,000 (~US$68,000) in grants to each enterprise over the first year, followed by interest-free loans for qualifying applicant during the subsequent year.

The 2001 National Biotechnology Strategy in South Africa was the key policy instrument that highlighted the need to capture the commercial opportunities offered by biotechnology. The strategy led to the establishment of Biotechnology Regional Innovation Centers (BRICS), the main government initiatives in biotech development. The South African government had earmarked 450 million Rands ($58 million) from 2004–2007 for related initiatives [12].

Overall, governments in China, India, Brazil and South Africa have played a crucial catalytic role in spurring health product innovation within the private sector – albeit on a limited scale. While all of the countries studied have housed strong universities and research institutes for decades, to kindle innovative activities within the private sector has required more focused government policies. It is noteworthy that the mere adoption of a product patent regime in pharmaceuticals by China in 1993 (even prior to its adoption of the TRIPS agreement and its enforcement in 2001), by Brazil in 1997, and the expectation of the pending TRIPS enforcement in India in 2005 were not sufficient, on their own, to spur significant entrepreneurial investment into the development of novel health products. Rather, entrepreneurial commitment to innovation, as a trend, became noticeable concomitant with increased government support for innovation within the entrepreneurial sector. In our experience the majority of emerging market companies that undertake innovation projects, with the objective to develop novel products, have benefited from governmental assistance. The more recent and targeted government policies attempt to increase entrepreneurial commitment to innovative R&D by direct subsidization of these efforts and/or through indirect measures. The latter mechanisms include building of shared R&D infrastructure, particularly in China, enhancing entrepreneurial access to public-sector R&D resources in Brazil, support of technology-focused venture capital funds most notably in China and Brazil, and the creation of Biotechnology Regional Innovation Centers in South Africa. It remains to be seen whether governmental efforts in these nations will be of sufficient scale and duration to help galvanize the private investments necessary for a successful transition to innovative sectors.

Relatively high and fast-rising domestic consumption is undoubtedly contributing to the biopharmaceutical sector’s development in the key emerging markets studied. At the same time, how governments implement cost-containment and central procurement measures may have considerable implications for innovation within the sector – particularly for diseases that predominantly affect local, and other developing world, populations.

### Integration into the global innovation system

Four key trends point to the globalization of health innovation, with a growing integration of emerging market firms, particularly evident in India and China. These trends add to existing relationships between pharmaceutical MNCs and emerging market firms, which have, until recent years, been largely limited to contract manufacturing and marketing. First, as discussed previously, there is an increasing number of co-development partnerships between firms in China and India and their foreign counterparts. Through these partnerships, companies in emerging economies have developed capabilities that have enabled independent basic research and commercialization. Second, large multinational corporations finance some innovation projects within emerging market firms in return for future development/marketing rights. These relationships rely on development of IP generated and/or owned by the emerging market partner. This practice has augmented the capabilities of emerging-economy partners with crucial relationships that subsequently enable independent commercialization and marketing activities. Third, the emergence of R&D services-based businesses that provide sophisticated and cost-effective research, development, and manufacturing should further augment innovative capabilities of the sectors as a whole at a time when the independent innovative capacity of multinational companies may be waning. Fourth, another emerging trend is the acquisition of domestic biopharmaceutical firms by foreign entities and the growing presence of the former in other nations. Examples of the first include recent acquisition of: India’s Shantha Biotechnics (Hyderabad) and Brazil’s Medley (São Paulo) by Sanofi Aventis (Paris, France), Piramal Healthcare (Mumbai) by Abbot Laboratories (Abbott Park, IL), India’s Ranbaxy by Takeda (Daiichi Sankyo, Tokyo), South Africa’s Vision Biotech (Cape Town) by Inverness Medical Innovations, Inc. (Waltham, MA), and China’s Guangdong Techpool Bio-Pharma (Guangzhou) by the Swiss drug maker Nycomed (Zurich, now part of Osaka-based Takeda Pharmaceutical Co.). Select emerging market firms have also ventured to expand their global presence through acquisitions or setting up subsidiaries. For instance, China’s CapitalBio Corporation has subsidiaries in San Diego (USA) and Hong Kong, Piramal Healthcare has a subsidiary in Canada (Torcan Chemical Ltd., Aurora). The above trends, as well as domestic industry consolidation serve to augments capabilities and enhance specialization, which makes indigenous enterprises more competitive globally.

Whether integration into the global system, on balance, proves beneficial to the emerging markets, or other developing nations, remains an open question. The suggestion here is merely that this phenomenon is indeed taking shape and in doing so is creating new opportunities – as well as new challenges – for emerging market entrepreneurs.

## Conclusions

This study shows that while China, India, Brazil and South Africa vary in certain respects, entrepreneurs in each of these countries aspire to become innovative in biopharmaceuticals, and have made inroads against this objective. Regardless of geography, these entrepreneurs are affected by similar forces and often respond in analogous ways. There are important similarities in national approaches to: enhancing innovative capabilities, breaking down innovation barriers, financing innovative activities often through innovative business models, collaborating with international firms, profiting from intellectual property, and to facing global competition. The commonalities highlight that biopharmaceutical firms in the China, India, Brazil and South Africa are influenced by forces emanating, in part, from industry globalization. Consequently, their responses carry implications for the global industry as a whole.

Our research shows that the trajectories of companies in the developed and emerging economies are becoming increasingly intertwined. Few of the firms we studied planned on developing novel therapeutics for global consumption fully on their own because they do not have the necessary technical and financial resources and cannot tolerate the associated investment risks. As a result, promising leads in development within the emerging market firms will likely find a home within the pipelines of major global players, or be developed through collaborative arrangements with other firms. Enhanced innovative capabilities in the emerging markets, the associated cost arbitrage, and improved IP protection regimes are increasingly facilitating outsourcing of R&D activities from pharmaceutical MNCs to emerging market firms. Yet many of the companies we studied also aspired to global competitiveness within an element of the value chain. The new pattern that seems to be emerging is a globally disintegrated drug development value chain with greater participation of the emerging markets at different stages of activity. This is not to suggest that this evolution will reduce diversity in business models. Indeed the opposite is likely to be true as companies carve out specific niches within an expansive, global value-chain.

In summary, the ultimate outcome of each country’s collective activity is likely to be shaped as much by global factors as local conditions. It is exceedingly important to realize that success in biopharmaceutical innovation is about the hard work of adapting scientifically, nationally and globally all at the same time. The implication is that countries that can best understand this complexity and situate their own strengths vis-à-vis the broader global value chain will be most likely to achieve sustainable competitive advantage. It is also possible that the globalization of biopharmaceutical innovation will have deleterious effects on domestic industries in the emerging markets, and on access to health technologies in the developing world. Failure to sufficiently adapt to new realities could, for instance, lock emerging market firms into low-value segments of the innovation value chain for years to come, and/or limit their growth prospects by preventing access to new technologies. These areas deserve further study and continued vigilance to minimize adverse impacts on emerging market industries and pharmaceutical access.

Notwithstanding above observations, recent developments in the emerging markets have the potential to change the global drug development model itself, with a promise to making it more productive and accessible. It offers the possibility for more cost-effective innovations and significant impacts on health systems everywhere. Technological upgrading within many of the existing pharmaceutical manufacturers in the emerging markets is leading to a more competitive global pharmaceutical generics market – leading to lower drug prices. At the same time, we are witnessing the birth of a highly integrated global industry when it comes to innovation – promising greater therapeutic choice and lower drug development costs. In this respect, emerging markets with greater economies of scale, in terms of domestic market size, and the scope of capabilities built over time appear to be in an advantageous position. The implication is that smaller countries such as South Africa with more limited biopharmaceutical purchasing power and skill-base to build on appear to be at a disadvantage vis-à-vis China and India. Similarly, historical dominance of the state sector and a host of other institutional factors contribute to dampening Brazil’s potential when it comes to health technology innovation. On the whole however, greater involvement of the entrepreneurial sectors in the emerging markets in health innovation holds considerable promise for patients everywhere.

## Abbreviation

AIDS, Acquired Immunodeficiency Syndrome; BRICS, Biotechnology Regional Innovation Centres (South Africa); DBT, Department of Biotechnology (India); FDA, Food and Drug Administration (U.S.); FINEP, Studies and Projects Funding Agency (Brazil); GAVI, Global Alliance for Vaccines and Immunization; GDP, Gross Domestic Product; HIV, Human Immunodeficiency Virus; IP, Intellectual Property; MNC, Multinational Corporation; NBRA, National Biotechnology Regulatory Agency; PAHO, Pan-American Health Organization; R&D, Research and Development; RMB, Renminbi or Yuan (Chinese Currency); SFDA, State Food and Drug Administration (China); SOE, State-Owned Enterprise; TB, Tuberculosis; TCM, Traditional Chinese Medicine; TRIPS, Trade Related Aspects of Intellectual Property Rights; UNICEF, United Nations International Children's Fund; VC, Venture Capital; WHO, World Health Organization.

## Competing interests

PAS is on the scientific advisory board of BioVeda China fund.

## Authors’ contributions

ASD, PAS and SEF participated in study design, sourcing of research funds and review/revisions of manuscript drafts. RR and SEF conducted interviews and drafted the manuscript. AM contributed to research design, and revisions to manuscript. All authors read and approved the final manuscript.

## Authors’ information

No additions required here.

## References

[B1] Lado AA Vozikis GS Transfer of technology to promote entrepreneurship in developing countries: An integration and proposed framework ET&P 1996 21 55 72

[B2] Daar AS Singer PA The Grandest Challenge: Taking Life-saving Science from Lab to Village 2011, Doubleday Canada

[B3] Trouiller P Olliaro P Torreele E Orbinski J Laing R Ford N Drug development for neglected diseases: a deficient market and a public-health policy failure Lancet 2002 359 9324 2188 94 10.1016/S0140-6736(02)09096-7 12090998

[B4] Kyle MK McGahan AM Investments in Pharmaceuticals Before and After TRIPS RESTAT Forthcoming

[B5] Lanjouw JO Cockburn IM New Pills for Poor People? Empirical Evidence after GATT World Dev 2001 29 2 265 89 10.1016/S0305-750X(00)00099-1

[B6] Frew SE Liu V Singer PA A Business Plan To Help The ‘Global South’ In Its Fight Against Neglected Diseases Health Aff 2009 28 6 1760 10.1377/hlthaff.28.6.1760 19887417

[B7] Rezaie R Singer PA Global health or global wealth? Nat Biotechnol 2010 28 9 907 9 10.1038/nbt0910-907 20829824

[B8] Marshall C Rossman G Designing Qualitative research 1999 3 Sage Publications, Thousand Oaks

[B9] Rezaie R Frew SE Sammut SM Maliakkal MR Daar AS Singer PA Brazilian health biotech–fostering crosstalk between public and private sectors Nat Biotechnol 2008 26 6 627 44 10.1038/nbt0608-627 18536682

[B10] Frew SE Sammut SM Shore AF Ramjist JK Al-Bader S Rezaie R Daar AS Singer PA Chinese health biotech and the billion-patient market Nat Biotechnol 2008 26 1 37 53 10.1038/nbt0108-37 18183014 PMC7096943

[B11] Frew SE Rezaie R Sammut SM India’s health biotech sector at a crossroads Nat Biotechnol 2007 25 4 403 17 10.1038/nbt0407-403 17420744

[B12] Al-Bader S Frew SE Essajee I Small but tenacious: South Africa’s health biotech sector Nat Biotechnol 2009 27 5 427 45 10.1038/nbt0509-427 19430446

[B13] Henderson R Orsenigo L Pisano G Mowery DC, Nelson RR The pharmaceutical industry and the revolution in molecular biology: Interactions among scientific, institutional and organizational change, In Sources of industrial leadership: studies of seven industries 1999 Cambride University Press, Cambridge 267 311

[B14] Hafner T Popp D China and India as suppliers of affordable medicines to developing countries, National Bureau of Economic Research Working Paper No. 17249 NBER 2011 [http://www.nber.org/papers/w17249]

[B15] Chaudhuri S Multinationals and Monopolies; Pharmaceutical Industry in India After TRIPS 2011, [http://apps.who.int/medicinedocs/en/m/abstract/Js19026en]

[B16] Chittoor R Ray S Aulakh PS Sarkar MB Strategic responses to institutional changes: ‘Indigenous growth’ model of the Indian pharmaceutical industry JIM 2008 14 252 69

[B17] Natesh S Bhan MK Biotechnology sector in India: strengths, limitations, remedies and outlook Curr Sci 2009 97 2 157

[B18] Milstien JB Gaul P Kaddar M Access to vaccine technologies in developing countries: Brazil and India Vaccine 2007 25 44 7610 9 10.1016/j.vaccine.2007.09.007 17913312

[B19] Li X The Impact of Higher Standards in Patent Protection for Pharmaceutical Industries under the TRIPS Agreement - A Comparative Study of China and India World Econ 2008 36 1367 82 [http://www.wider.unu.edu/publications/working-papers/research-papers/2008/en_GB/rp2008-36/]

[B20] Zhou EY Vaccine Development in China Biopharm Int 2007 20 4 2 5 [http://www.bioplanassociates.com/publications/articles/VACCINES_China_Zhou_Apr07_ChinaB.pdf]

[B21] Pereira FB A Biominas Study of the Brazilian Biotechnology Companies 2007,

[B22] Zhou P Leydesdorff L The emergence of China as aleading nation in science Res Policy 2006 35 1 83 104 10.1016/j.respol.2005.08.006

[B23] Science and Engineering Indicators, 2010. A National Science Board Publication, [www.nsf.gov/statistics/seind10)]

[B24] Simon DF Cao C China’s Emerging Technological Edge: Assessing the Role of High-end Talent 1999 Cambridge University Press, Cambridge

[B25] Weisburst S Scherer F Economic Effects of Strengthening Pharmaceutical Patent Protection in Italy Int’l Rev Ind Prop & Copyright L 1995 26 6 1009 1024

[B26] La Croix S Kawaura A Product patent reform and its impact on Korea’s pharmaceutical industry Int Econ J 1996 10 1 109 124

[B27] Kortum S Lerner J What is behind the recent surge in patenting? Res Pol 1999 28 1 1

[B28] Hall B The patent paradox revisited: an empirical study of patenting in the US semiconductor industry, 1979–1995 RAND J Econ 2001 32 1 101 128 10.2307/2696400

[B29] Sakakibara M Do stronger patents induce more innovation? Evidence from the 1988 Japanese patent law reforms RAND J Econ 2001 32 1 77 100 10.2307/2696399

[B30] Kanwar S Evenson R Does intellectual property protection spur technological change? Oxford Econ Pap 2003 55 2 235

[B31] Moser P How do patent laws influence innovation? Evidence from nineteenth-century world’s fairs 2005 95 4 1214 1236

[B32] Levin R Klevorick A Nelson R Winter S Gilbert R Griliches Z Appropriating the returns from industrial research and development Brookings PapEcon Act 1987 3 783 831

[B33] Scherer F Weisburst S Economic effects of strengthening pharmaceutical patent protection in Italy Int’l Rev Ind Prop & Copyright L 1995 26 6 1009 1024

[B34] Noguès J Social costs and benefits of introducing patent protection for pharmaceutical drugs in developing countries Dev Econ 1993 31 1 24 53

[B35] Ward M Dranove D The vertical chain of research and development in the pharmaceutical industry Econ Inquiry 1995 33 70 70 10.1111/j.1465-7295.1995.tb01847.x

[B36] Finkelstein A Static and Dynamic Effects of Health Policy: Evidence from the Vaccine Industry QJE 2004 119 2 527 564 10.1162/0033553041382166

[B37] Chadha A Oriani R R&D market value under weak intellectual property rights protection: the case of India Scientometrics 2010 82 1 59 74 10.1007/s11192-009-0042-x

[B38] Zhang YP Deng MM Enforcing pharmaceutical and biotech patent rights in China Nat Biotechnol 2008 26 11 1235 40 10.1038/nbt1108-1235 18997760

[B39] Rongping M The innovation risk of enterprises need to be shared via incentive policies and the development of public infrastructure to support innovation UNESCO Science Report 378 378 99 [http://www.unesco.org/new/en/natural-sciences/science-technology/prospective-studies/unesco-science-report/unesco-science-report-2010/]

[B40] de Brito Cruz C Chaimovich H Industrial R&D still suffers from a lack of government support, even though the situation has improved radically over the past eight years UNESCO Science Report 2010 103 103 21 [http://www.unesco.org/new/en/natural-sciences/science-technology/prospective-studies/unesco-science-report/unesco-science-report-2010/]

[B41] Mani S The main challenge facing the country will be to improve both the quality and quantity of S&T Personnel UNESCO Science Report 2010 362 77

[B42] OECD Report; Science, Technology and Industry Outlook 2008, [http://www.oecd.org/document/19/0,3746,en_2649_37417_46680723_1_1_1_37417,00.html]

[B43] UNESCO Science Report 2010, [http://www.unesco.org/new/en/natural-sciences/science-technology/prospective-studies/unesco-science-report/unesco-science-report-2010/]

